# Visual Attention-Related Processing: Perspectives from Ageing, Cognitive Decline and Dementia

**DOI:** 10.3390/brainsci11020206

**Published:** 2021-02-09

**Authors:** Claire J. Hanley, Andrea Tales

**Affiliations:** 1 Department of Psychology, Swansea University, Swansea SA2 8PP, UK; 2 Centre for Innovative Ageing, Swansea University, Swansea SA2 8PP, UK; A.Tales@swansea.ac.uk

Regarded as a defining factor in resource management, it is widely accepted that visual attention and related processing will deteriorate, in a global fashion, across the lifespan and produce detrimental consequences for environmental interactions. Disproportionate detriments in such functions are also proposed to arise in the context of a range of age-related disorders. However, the nature and extent of such losses, the precise mechanisms that underlie these changes, and the origin of deficits in task performance that are used to assess this multicomponent process are still under debate. This Special Issue, comprising studies spanning healthy ageing, cognitive impairment, and dementia, represents a body of literature that fundamentally advances these points of discussion.

Contrary to previous findings and prior assumptions, the research documented herein attests to a large degree of preserved task performance in ageing. First, with regard to the role of attention in working memory (WM), by simultaneously assessing participants’ ability to filter and ignore distracting stimuli, Maniglia and Souza [1] separate the roles of encoding and maintenance in the first study to do so in an older adult sample. Through Bayesian analysis, the authors establish a lack of age-related deficit with regard to both components while also providing evidence for similar effects of memory load and distraction across the adult lifespan. These findings signify that older participants had no significant problems allocating sufficient attentional resources to manage visual WM representations compared to their younger counterparts. Although they require extended time to engage attention, older adults were just as efficient at encoding relevant stimulus features. In this instance, the findings suggest that visual WM performance cannot be explained by deficits in attentional control, as is often speculated. Therefore, age-related differences should not be assumed in this context, and where they are observed, should not be automatically presumed to arise as a result of deficient inhibition of distracting items or irrelevant stimulus attributes.

Henderson et al. [2] also identified similar strategies between younger and older adults, suggesting the ability to flexibly manage attention allocation to support visual WM performance is generally maintained across the lifespan. Each group successfully utilised spatial cues to modify attention and ignore distractors when cue validity was completely certain. However, the presence of this implied age-related functional integrity varied depending on the priority assigned to the stimulus. Accordingly, the authors suggest the successful updating of details that are no longer considered relevant is highly influenced by the extent to which older adults have to divide their attention under high demands. The most compelling models of the findings indicated that the high proportion of nontarget errors demonstrated by older adults could be attributed to the lasting impression of the priority assigned at the encoding stage. Therefore, older adults failed to inhibit the no-longer-relevant yet originally prioritised item because initially low-priority items could not be retrieved when resources were in short supply. This proposal has valuable applications in assessing the efficacy of visual WM in older adults, which could be predicted on the amount of resources allocated to a stimulus and may translate to other executive tasks. On the basis of this evidence, where age-related deficits in visual WM are observed (most likely due to priority biases), they should not be attributed to poor strategy formation but, instead, the inability to engage adequate inhibitory control.

Further extending the discussion of integrity of function to those with mild cognitive impairment, Revie et al. [3] highlight an absence of age-related information processing deficits via a drift rate model applied to choice reaction time data. Healthy older adults were found to be more cautious with regard to decision making but did not demonstrate differences in drift rate to those with mild cognitive impairment in Lewy body disease (MCI-LB). Furthermore, this study addresses the debate as to whether the origin of visual hallucinations in those with MCI-LB can be attributed to perceptual or attentional deficits, an important consideration, as hallucinations impact upon cognitive decline and quality of life. The authors conclude that while not significantly impaired, all patients exhibited a slowing of sensory processing, whereas those who experience hallucinations also displayed executive deficits in decision-making. Such knowledge will undoubtedly pave the way for further exploration into the onset of hallucinations as well as targeted interventions to alleviate their detrimental consequences.

Across the lifespan, and between states of health and disease, the extent to which individuals are able to adequately manage attentional allocation remains contested. Extending the debate on the control of resources to the domain of divided attention, Barel and Tzischinsky [4] investigate object–location memory, an ability crucial to everyday functioning. A study with an incredibly novel focus, the authors provide a targeted demonstration of how top-down attention can determine memory performance. Addressing the role of attentional and executive functioning in incidental compared to intentional episodic memory encoding, they demonstrate superior performance in the incidental condition. Furthermore, vigilance correlated with memory performance scores in the incidental condition only, suggesting that under increased cognitive load sustained attention influenced incidental but not intentional encoding.

Additionally, Polden et al. [5] compared the ability of healthy younger and older adult controls to those with MCI and Alzheimer’s disease (AD) to successfully disengage and refocus attention, while incorporating eye-tracking to facilitate measures of the “gap effect”. With regard to the influence of age, in contrast to their younger counterparts, older participants demonstrated slower reaction times and greater difficulty in disengaging attention, in addition to a general slowing in prosaccades. Perhaps surprisingly, performance and prosaccades were similar in comparison to the patient groups, providing insight into preservation of the “gap effect” in states of cognitive impairment. The authors established that only in the MCI group were longer reaction times correlated with poorer working memory, inferring that such scores—reflecting attentional disengagement—could act as a valuable metric during what is considered to be a transition period to AD for some individuals. The research emphasises that while the superior colliculus appears to remain intact, executive regions of the network such as the anterior cingulate, which are crucial for inhibitory control, are likely to be affected early on in the disease process. Indeed, there may be important lessons to be learned from this study to focus on identifying the subtleties of preserved function instead of fixating on targeting deficits, while also addressing individual differences in factors such as ethnicity. These considerations could have important implications with regard to the assessment of attention-related processing and associated approaches to enhance existing function.

Building on the role of participant characteristics, Torrens-Burton et al. [6] explored the occurrence and origin of age-related attentional control deficits to a complex yet naturalistic task-switching paradigm. Investigating a range of variables (including subjective memory function, cognitive reserve, gender, and education), the authors echo the sentiment of this collection of articles by demonstrating that while age can be inferred to influence speed, it seldom fundamentally alters proficiency. The ability to modulate attention to attend to a target and inhibit interference, under conditions of increasing difficulty brought about by greater task speed, can be considered significantly less efficient in older adulthood. However, age itself was not a contributing factor to performance accuracy, and while statistically slower than younger adults, older participants were no less precise. Scores reflecting general cognition (determined by the Montreal Cognitive Assessment, MoCA) but not age itself, were associated with speed of performance. MoCA performance can, therefore, be considered a potential marker of information processing capacity in the context of the task, but the overarching message is that attentional control does not appear to vary by age per se. Consequently, translating performance of a dynamic task to real-life demonstrations of such executive function (e.g., in the context of driving), the study implies that chronological age is unlikely to be a direct contributing factor in proficiency. Moving forward, it will be of extreme importance to identify the factors that underlie superior cognitive scores (as the MoCA results did not relate to perceived level of cognitive function or the other variables assessed as part of the research) if they are to prove informative.

The real-world insights advocated by the aforementioned study could produce incredibly valuable applications considering driving cessation is a major life transition, with implications for health and well-being. Huizeling et al. [7] enhance our understanding of the complexities of refocusing attention in such a naturalistic setting, presenting the first study to acquire task-related oscillatory data, using electroencephalography (EEG), from a subgroup of older adult participants to extend inferences based on simulated driving measures. The data support the pattern of age-related slowing of reaction times shown consistently in this collection of articles—in this instance, reflecting adjustments older adults make to their driving behaviour to accommodate deficient attentional control mechanisms. Consequently, the processing difficulties reported in abstract computer tests also appear to translate to such realistic settings. The incidence of slow reactions with increased age was established in several tasks: from the time to read road sings to that taken to indicate. Additionally, where attention was already engaged to monitor the behaviour of other drivers, it was more difficult for older adults to widen their focus to identify a relevant target among distractors. These behavioural findings were accompanied by age-related differences in theta and alpha power, which culminate to suggest that older, compared to younger, adults appear to be less able to engage the attentional resources required to switch focus and respond to dynamic changes in a flexible manner. The authors note that switching was affected by how predictable a stimulus was (corresponding to the findings outlined here [1,2]), yet the demands of driving are anything but predictable. Where older adults appear to adopt fundamentally different, and largely maladaptive, strategies during driving, this study provides pivotal evidence of the need for interventions that can facilitate safe driving behaviour, which will be particularly poignant past the point where the bounds of cognitive and neural compensation mechanisms are exceeded (in those most vulnerable to decline; e.g., states of cognitive impairment, lower cognitive reserve, as well as “old-old” age and beyond).

In recent years, non-invasive brain stimulation techniques (NIBS) have been heralded as compensation aids due to their ability to enhance neural plasticity. Use of these methods could, therefore, represent a powerful approach to sustaining cognition in ageing. However, beyond initial successes, the multitude of stimulation parameters available to researchers, especially those exploring transcranial direct current stimulation (tDCS), makes it extremely difficult to optimise protocols and maximise beneficial outcomes. Hanley et al. [8] address this quandary by systematically investigating stimulation duration, using a variation of the dynamic task-switching paradigm that features here [6]. Sham control stimulation failed to enhance performance, suggesting a lack of practice or placebo effects, whereas superior performance speed was documented following active tDCS. However, contrary to predictions, ten minutes of stimulation was shown to produce advantageous results when compared to the longer twenty-minute variant. The authors speculate that the success of the shorter intervention can likely be attributed to participant characteristics, which determine the status of grey and white matter integrity and resulting presence of cerebrospinal fluid. While the results of this study represent a hugely exciting prospect for on-going efforts to minimise cognitive decline, continued research is needed to better understand the action of NIBS techniques and the influence of individual differences if we are to fully harness their value.

Therein, this collection of articles represents cross-modality research at the cutting-edge of advances in the multifaceted domain of visual attention-related processing. Crucially, each contribution in this Special Issue attests to the ageing process being dynamic and not simply characterised by global deficits. Similar inferences can be made in the context of various forms of cognitive impairment (MCI, MCI-LB, AD), where elements of preserved function have also been identified in the articles presented here. These considerations are highly important as they have direct consequences for prospective interventions, with the presence of individual differences implying that a one-size fits all approach will fail to incorporate the complexities outlined in the showcased research. By addressing the integrity of function via a unique array of approaches, encompassing novel methodologies and innovative concepts, these studies ultimately culminate in improving (1) our understanding of age-related changes, (2) the identification of potential signatures of a range of disease processes, and (3) interventions to attenuate the impact of observed decline and maintain quality of life. With a firm emphasis on the development of naturalistic applications, which test the assumptions put forward by basic research, an increase in the type of investigations advocated here will ensure the real-life relevance of future endeavours.
